# Harmonization of late-life participation in cognitively stimulating activities across four cohort studies of cognitive aging

**DOI:** 10.1016/j.exger.2026.113069

**Published:** 2026-02-13

**Authors:** A.R. Zammit, A.L. Gross, L. Yu, L.L. Barnes, R.S. Wilson, D.A. Bennett

**Affiliations:** a Rush Alzheimer's Disease Center, Rush University Medical Center, Chicago, IL, USA; b Department of Psychiatry and Behavioral Sciences, Rush University Medical Center, Chicago, IL, USA; c Department of Epidemiology, Johns Hopkins Bloomberg School of Public Health, Baltimore, MD, USA; d Department of Neurological Sciences, Rush University Medical Center, Chicago, IL, USA

**Keywords:** Harmonization, Cognitive activities, Cognitive decline, cognitive resilience

## Abstract

**Objective:**

We harmonized self-reported survey data measuring late-life participation in cognitively stimulating activities across four cohort studies of cognitive aging.

**Methods:**

Data came from the Rush Memory and Aging Project (MAP), the Minority Aging Research Study (MARS), the Rush ADRC Clinical Core (Clinical Core), and the Religious Orders Study (ROS) in individuals free of dementia at baseline. We used an item-banking approach that leverages all available items assessing cognitively stimulating activities, including items common and unique across studies. We generated harmonized factors to summarize an individual's cognitive activity. We then validated the harmonized factor by testing its association with incident AD dementia, cognitive decline, and cognitive resilience i.e. cognitive decline after adjusting for common ADRD pathologic indices.

**Results:**

We included 4550 participants (MAP = 1977; MARS = 825; Clinical Core = 366; ROS = 1382); 75% were female, mean baseline age was 76.9 years (SD = 7.6) and mean education was 16 years (SD = 3.7). Confirmatory factor analysis models fit the data well in each study (comparative fit index ≥ 0.90, root mean square error of approximation ≤ 0.08, and standardized root mean residual ≤ 0.08). The harmonized factor score was associated with incident AD dementia (HR = 0.80, 95%CI = 0.74–0.86, p < 0.001), cognitive decline (est. = 0.005, SE = 0.002, p = 0.02), and cognitive resilience (est. = 0.011, SE = 0.004, p = 0.003).

**Conclusion:**

We statistically harmonized survey data measuring frequency of participation in cognitive activities across four studies of aging and subsequently validated the harmonized latent trait by linking it to ADRD outcomes. Findings demonstrate the utility of combining self-reported psychosocial survey data collected across multiple studies to thoroughly evaluate the impact of modifiable risk factors on later-life cognitive outcomes in heterogeneous populations.

## 1. Introduction

Frequent participation in cognitively stimulating activities is a key contributor of cognitive health in older adults (Stern and Munn, 2010). A large body of research suggests that frequent participation in cognitively stimulating activities, such as reading books and playing board games, may help build cognitive reserve by reducing the odds of Alzheimer's disease (AD) dementia, maintaining higher cognitive function and decelerating cognitive decline in later life (Ferreira et al., 2015; Gow et al., 2017; Staff et al., 2018; Mitchell et al., 2012; Verghese et al., 2003; Scarmeas et al., 2001).

In our prior work, we showed that higher frequency of participation in cognitively stimulating activities is associated with lower odds of AD dementia (Wilson et al., 2003a; Wilson et al., 2007), and with a slower rate of cognitive decline (Wilson et al., 2007), even after we control for common Alzheimer's Disease and related dementias (ADRD) neuropathologic indices (Wilson et al., 2013), indicating higher cognitive resilience afforded by these activities. However, these studies were conducted in separate cohort studies and each used slightly different cognitive activity surveys.

In an effort to link psychosocial survey data that reflect modifiable reserve and resilience measures we followed Maelstrom Research guidelines (Fortier et al., 2017) to statistically harmonize two different surveys indicating frequency of participation in cognitively stimulating activities assessed at enrollment across four studies of aging – the Rush Memory and Aging Project (MAP), the Minority Aging Research Study (MARS), the Rush ADRC Clinical core (Clinical Core), and the Religious Orders Study (ROS) in individuals who were free of dementia at baseline. We validated the harmonized latent factor by testing its associations with incident AD dementia, with cognitive decline, and with cognitive resilience i.e. cognitive decline after adjusting for nine common ADRD neuropathologic indices, in a subset with postmortem data.

## 2. Methods

### 2.1. Study participants

Participants were community-living older adults enrolled in one of four ongoing clinical-pathologic cohort studies of aging: MAP; MARS; Clinical Core; ROS. Enrollment began in 1994 for ROS, in 1997 for MAP, in 2004 for MARS, and in 2008 for Clinical Core. ROS enrolls participants who are older nuns, priests, and religious brothers recruited across the USA (Bennett et al., 2018). MAP enrolls participants who live in northeastern Illinois (USA) and are recruited from personal accommodations, subsidized housing, and retirement facilities (Bennett et al., 2018). Both MARS and Clinical Core enroll only African American individuals living in the greater Chicago metropolitan area (Barnes et al., 2012). Participants consent to annual clinical evaluations and sign an informed consent and Anatomic Gift Act for brain donation at death (optional for MARS and Clinical Core) (Bennett et al., 2018; Barnes et al., 2012). Participants who met dementia criteria at baseline (MAP = 100, MARS = 18, Clinical Core = 23, ROS = 98) were excluded. All studies were approved by an Institutional Review Board at Rush University Medical Center.

### 2.2. Measure for harmonization: late-life cognitively stimulating activities

Participants in the MAP, MARS and Clinical Core studies were given a nine-item survey at baseline in which they reported frequency of participation in various mentally stimulating activities over the prior year including reading books, magazines, newspapers, going to the library, visiting museums, writing letters and playing games. Frequency of participation in each activity was rated on a 5-point Likert scale as follows: (5) every day or about every day; (4) several times a week; (3) several times a month; (2) several times a year; (1) once a year or less.

Participants in the ROS study were given a seven-item survey which also reported frequency of participation in seven common cognitively stimulating activities, 5 of which had similar question prompts with the other studies. Frequency of participation in each activity was also rated on a 5-point Likert scale with the same response choices as those for the MAP, MARS, and Clinical Core.

A full description of the items is provided in S. Table 1. Five overlapping items across the 4 studies are highlighted in green, and six non-overlapping items in gray. Overlapping items include questions relating to frequency of i) reading newspapers, ii) reading magazines, iii) reading books, iv) playing games, such as cards, checkers, crosswords, or other puzzles or games, in the past year. The final overlapping item related to frequency of visiting museums over the past 10 years in MAP, MARS & Clinical Core, and without a time-period in ROS.

### 2.3. Measures for external validation

We hypothesized that higher levels of later-life cognitively stimulating activities correlate with lower odds of AD dementia, better baseline global cognition, less steep cognitive decline, based on previously established associations in some of the individual studies (Wilson et al., 2007; Wilson et al., 2013). To evaluate discriminant validation evidence, we further hypothesized the measure would not be related to 9 common ADRD neuropathologic indices.

### 2.4. Diagnosis of AD dementia

AD dementia was diagnosed by an experienced clinician using criteria of the joint working group of the National Institute of Neurologic and Communicative Disorders/Stroke/AD and Related Disorders Association (McKhann et al., 1984; Bennett et al., 2002; Wilson et al., 2002a). These criteria require a history of cognitive decline and evidence of impairment in at least two domains of cognitive function, one of which being memory (Bennett et al., 2002).

### 2.5. Assessment of global cognition

Cognitive ability was assessed with a battery of 21 neuropsychological tests, as previously described (Wilson et al., 2002b; Wilson et al., 2004). 19 of the individual test scores were transformed into z-scores using baseline means and standard deviations of the four studies. A composite measure of global cognitive function was constructed by averaging the z-scores across the 19 tests, and a higher score represents better cognitive function.

### 2.6. Postmortem neuropathology

Neuropathologic data collection and assessment were performed blinded to all clinical and cognitive data, and followed a standard protocol for tissue preservation, tissue sectioning, and quantification of pathologic findings (Schneider et al., 2006; Bennett et al., 2006) as previously described (Schneider et al., 2009a). The cerebral hemispheres were coronally cut into 1-cm slabs. One hemisphere was fixed in 4% paraformaldehyde for histologic evaluation, and the other hemisphere was frozen. Tissue blocks from predetermined regions were dissected, embedded in paraffin, and cut into 6 μm sections (Schneider et al., 2009b). Measured indices of ADRD pathologies included (1) Alzheimer's disease pathology, (2) hippocampal sclerosis, (3) TAR DNA-binding protein 43, (4) Lewy bodies, (5) macroinfarcts, (6) microinfarcts, (7) cerebral amyloid angiopathy, (8) atherosclerosis, and (9) arteriolosclerosis (Schneider et al., 2012).

### 2.7. Approach

#### A. Pre-statistical harmonization

**Fig. 1.**
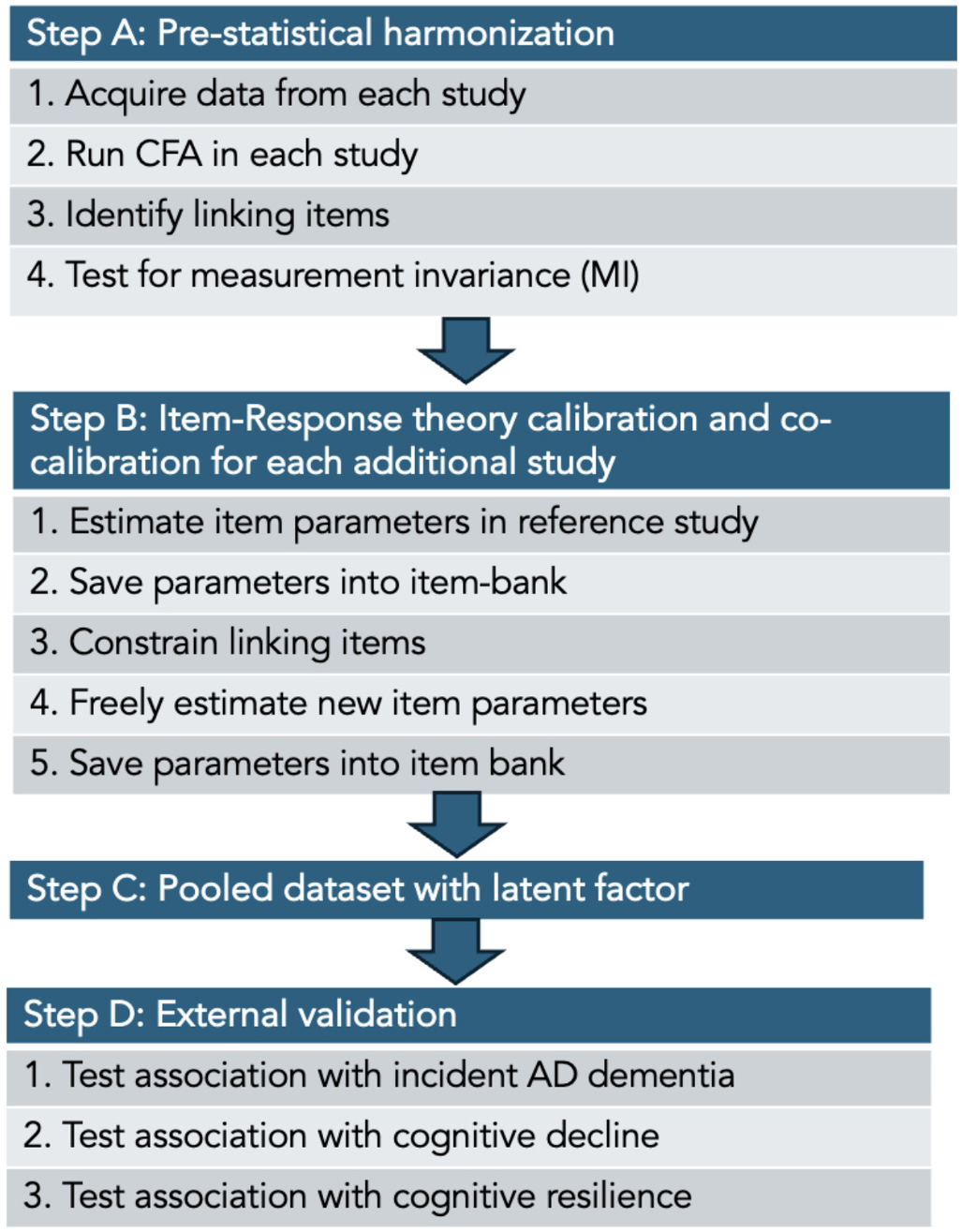
Key steps taken in this harmonization study.

Pre-statistical harmonization is a key first step in which comparable items are identified, described and analyzed (Griffith et al., 2013). We outline previously established key harmonization steps (Gross et al., 2023; Mukherjee et al., 2023) that we adopted in our study in Fig. 1.

##### i) Acquire data from each study.

We first acquired item-level data from each of the studies and examined correlations among items (S. Tables 2–4).

We began our item-banking by combining cross-sectional data from the four studies: MAP (n = 1977), ROS (n = 1382), MARS (n = 825), and Clinical Core (n = 366). We combined MARS and Clinical Core since both studies enroll older African Americans from the same geographical region and the participants share similar demographics (MARS: age = 73.4 SD = 6.2; 77% female; years of education = 14.9, SD = 3.4; Clinical Core: age = 72.8, SD = 6.3; 82% female; years of education = 14.7 SD = 3.1). Each item was treated as an ordinal variable. We used data from the baseline visit for harmonization. Missing data were sparse (0.01% in ROS; 0.03% in MAP; 2.7% in MARS & Clinical Core).

##### ii) Confirmatory factor analyses of late-life cognitively stimulating activities in each study

To empirically assess that similar activity items fit well across studies before imposing assumptions about linking them between studies, we conducted confirmatory factor analysis(CFA) in MPlus using robust weighted least squares (WLSMV) estimator (Beauducel and Herzberg, 2006; Flora and Curran, 2004) since we had ordinal indicators. WLSMV is useful to estimate parameters when using ordinal data that may not be normally distributed such as in Likert scales and provides robust parameter estimation when normality assumptions may be violated. Separate CFAs were conducted in MAP, MARS & Clinical Core, and ROS. Absolute model fit indices included root mean square of approximation (RMSEA, good fit ≤ 0.05), comparative fit index (CFI, good fit > 0.95), and standardized root mean residual (SRMR, good fit <0.080).

Factor loadings between 0.30 and 0.90 suggest that items are meaningfully related to the underlying trait, and homogeneity among loadings implies no single item is overwhelming the measurement model (Stefana et al., 2024). Importantly, criteria for CFA fit must also depend on theoretical consideration (Brown, 2015; McDonald, 2013). For instance, while items related to reading e.g. newspaper, magazines, and books, might be highly correlated and have high factor loadings, other items e.g. playing board games or visiting museums might not be expected to load as highly, yet these activities still entail active cognitive engagement.

##### iii) Identification of linking items across studies

Co-calibration requires either same people taking different tests, or different groups of people sharing common items. In this study, we had common items across the MAP, MARS & Clinical Core, and ROS studies. We identified five potentially common items: i) read newspapers, ii) read magazines, iii) read books, iv) play games, v) visit museums. We paid close attention to the stem question prompt as well as response options, which varied somewhat across the studies. Most notably, the “visit museums” question asked MAP and MARS & Clinical Core, “In the last ten years, how many times did you visit a museum?”, and asked ROS participants, “How often do you go to museums?”. The response options to this question differed too, from 1 = “never”, 2 = “1–2 times”, 3 = “3–9 times”, 4 = “10–19 times”, 5 = “more than 20 times” for MAP and MARS & Clinical Core, and 1 = “once a year or less”, 2 = “several times a year”, 3 = “several times a month”, 4 = “several times a week”, 5 = “every day or almost every day” for ROS. Additionally, while non-linking question prompts varied slightly across MAP, MARS & Clinical Core and ROS, linking items were all on the same Likert scale across studies. We ensured that stem question prompt of each linking item was comparable across studies. For example, as detailed in S. Table 1, to the stem question, “How often do you read newspapers” the possible response across all studies was 1 = almost every day, 2 = several times a week, 3 = several times a month, 4 = several times a year or 5 = once a year or less; this item was recoded so that higher scores reflect more late-life cognitively stimulating activities.

##### iv) Testing for measurement invariance among linking items

To ensure that the linking items are measuring the underlying latent trait in the same way across the studies, we tested for measurement invariance among the linking items by evaluating whether the same indicators loaded on the common factor in each study (configural invariance), whether the factor loadings are equivalent in each study (metric invariance; analogous to testing whether random errors in each item are comparable among studies, adjusting for the underlying latent trait), and whether the item thresholds are the equivalent across studies (scalar invariance; analogous to testing for systematic differences or biases in item location across cohorts, adjusting for the underlying latent trait). The result of testing for invariance was to quantitatively confirm that theoretically defined linking items across cohorts reflected the underlying trait equivalently across the studies.

#### B. Item-Response theory calibration of the cohort studies by confirmatory factor analyses and co-calibration for each additional study

We statistically harmonized scores across the studies using an item banking approach (Vonk et al., 2022). To carry out the item-banking procedure, we used the MAP study as the reference since this cohort is larger than the others. Thus, model parameters were first estimated and saved from a confirmatory factor analysis using data from MAP. By convention, the mean and variance of the latent trait were set to 0 and 1, respectively.

We then conducted CFA in each study, by fixing model parameters for linking items to the corresponding values from the reference study. The confirmatory factor analysis models estimated two relevant parameters for each item: factor loadings and thresholds. Factor loadings describe the strength of association between the item and the underlying latent factor. Thresholds reflect the average level of the underlying trait at which the item is most discriminating.

Following the CFA on MAP, we first repeated the analysis for MARS & Clinical Core. While MARS & Clinical Core's items were all common with MAP, and all cohorts show similar fit and loadings, we only fixed the model parameters of the 4 linking items (items 1–4, shaded green in S.Table 1) by using estimates derived from MAP and freely estimated the parameters of the non-linking items (item 5, shaded orange, and items 6–9 shaded gray in S.Table 1). Then, we conducted CFA for ROS by fixing the model parameters of the linking items using the estimates derived from MAP, while the parameters of the non-linking terms were freely estimated. Thus, from the item banking procedure, parameters for 5 non-linking items in MARS & Clinical Core (items 5–9 in S.Table 1) and 3 non-linking items that were present in ROS (items 5, 10–11 in S. Table 1) were freely estimated, then saved in the item bank. In the final step we conducted a confirmatory factor analysis using the pooled sample of all participants where all the parameters were fixed to using the estimates previously stored in the item bank.

#### C. Harmonization: Pooled dataset with latent factor

From the final co-calibration model, we extracted all item parameters (loadings and thresholds). These values populated our item bank. A final score-generating model pooled all participants using all previously estimated parameters. Factor scores were generated using expected a posteriori (EAP) estimation via maximum likelihood with robust standard errors (MLR) in Mplus. We used MLR for pooled scoring because it allows for full information maximum likelihood estimation which supports generating harmonized factor scores on a common metric across cohorts while accommodating incomplete data patterns across studies during harmonization.

#### D. External Validation

We then assessed the validity of the harmonized factor score as a measure of later-life cognitively stimulating activities among older adults in the US, estimated from the score-generating IRT model, by examining its associations with incident AD dementia, cognitive decline, brain pathologies, and cognitive decline controlling for the pathologic indices, reflecting resilience. We used proportional hazards models to examine the association of the harmonized factor score for late-life cognitive activity with the hazard ratio of developing incident AD dementia, adjusting for baseline age, sex, race, and education, using this larger pooled cohort. We also used accelerated failure-time (AFT) models to estimate mean time to AD dementia, adjusting for the same covariates.

We used linear mixed-effects (LME) models to test the associations of the harmonized factor score with baseline level and rate of cognitive change. The primary outcome was a longitudinal composite measure of global cognition, adjusting for the same covariates.

In a third set of regression models we examined the relation of the harmonized factor score to 9 common neuropathologies.

In a final validation analysis, we did another set of LME models examining whether the harmonized factor score was related to level of cognition or rate of cognitive change before death, adjusting for age at death, sex, race, education, and nine common ADRD neuropathologic indices. We estimated these models in the whole sample and in individual cohorts to determine whether the association of the harmonized factor score for later-life cognitive activities with the cognitive outcomes are reflective of the association of the individual late-life cognitive activity measures with these outcomes within each cohort.

CFA models were estimated with MPlus software (Muthén, 2017) (Version 8.11), R software (R: A Language and Environment for Statistical Computing, 2024) (Version 4.5.1), SPSS (Version31) and SAS software were used for data management and external validation analyses.

## 3. Results

### 3.1. Study participants

A total of 4550 participants were included. The pooled sample was 75% female; on average participants were almost 77 years old at enrollment, highly educated (pooled mean education = 16 years), and followed up for an average of 8 years (SD = 6) (Table 1). Mean scores on each item stratified by cohort are presented in S. Table 5.

### 3.2. Confirmatory factor analyses in each study

Fit of CFA models of late-life cognitively stimulating activities in each study was excellent (MAP: CFI = 0.960, RMSEA = 0.047, SRMR = 0.029; MARS&Clinical Core: CFI = 0.947, RMSEA = 0.050, SRMR = 0.032, ROS: CFI = 0.943, RMSEA = 0.032, SRMR = 0.022). Most standardized factor loadings were > 0.3, indicating that each item is meaningfully related to the underlying factor (Brown, 2015) (S.Table 6). The average factor loading tended to be lowest in the ROS (average = 0.3), however the model still fit the data well. Since low loadings can still provide information on the underlying latent trait, we plotted item information curves (IIC) for the items in each study to illustrate the range of information of individual items. As can be seen in S. Fig. 1, items with low factor loadings in ROS (anchor items: “*How often do you read newspapers?”*, and *“How often do you read magazines”?*) contributed the most information corresponding to the peaks of the item curves. At the same time, while anchor item: “*How often do you play games?”* exhibited low IICs across all studies, indicating minimal discrimination on the latent trait, it was conceptually similar across studies.

**Fig. 2.**
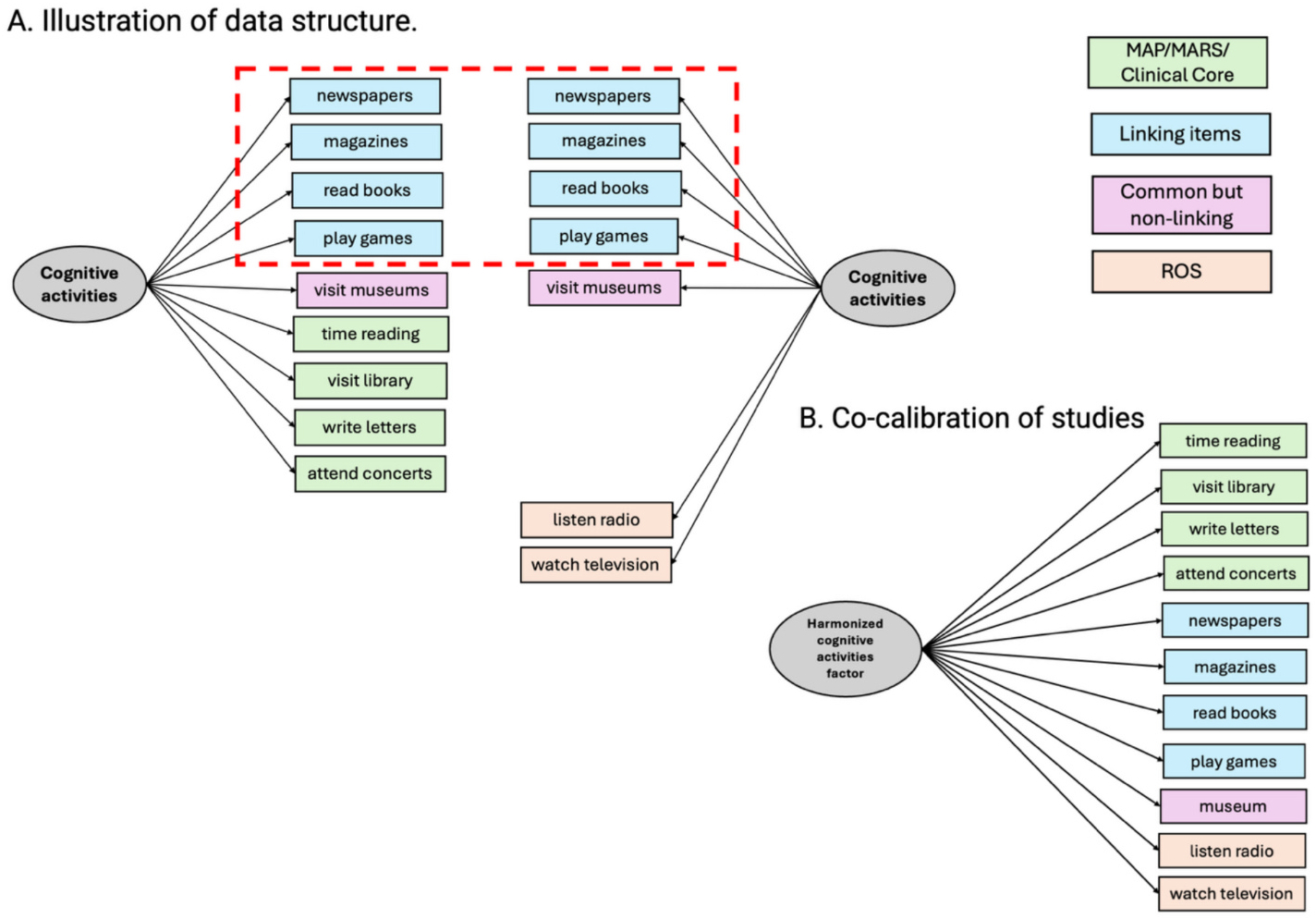
(A) Illustration of data structure and (B) co-calibration of studies. **(A)** Data illustrating survey items from two studies at a time, with the linking items in the red dotted box. The Rush Memory and Aging Project (MAP), the Minority Aging Research Study (MARS), and the Rush ADRC Clinical Core (Clinical Core), had a similar data structure, displayed on the left, and the Religious Orders Study (ROS)'s data structure is displayed on the right. We used the Rush MAP as the reference cohort to generate starting values for the linking items. Non-linking items for the MARS & Clinical Core and the ROS studies were freely estimated. (**B)** Co-calibration of pooled items across the four studies.

Of the 11 items, we compared two sets of models for measurement invariance, one with five linking items (newspapers, magazines, books, games, museums) and one with four linking items (newspapers, magazines, books, games). We dropped the museums item because it violated the monotonicity assumption on a common latent trait due to distinct scale semantics in the surveys across the cohorts as noted above. Specifically, the item in MAP, and MARS & Clinical Core captured cumulative exposure over a fixed period i.e. *in the past 10 years, how many times* did you visit a museum?, while the item in ROS it captured frequency i.e. “*How often* do you go to museums?”. Relatedly, the lifestyles of participants from the ROS might involve fewer opportunities to attend museums due to religious constraints and since many live in rural areas without nearby museums. Indeed, the five-item linking model established good fit for the configural model (CFI = 0.930, TLI = 0.860, RMSEA = 0.060, SRMR = 0.030); however, the four-item model had slightly better overall fit (CFI = 0.967, TLI = 0.929, RMSEA = 0.049, SRMR = 0.020). Moreover, the change in CFI from the configural and metric models for the 4-item model (ΔCFI = 0.003), but not the 5-item model (ΔCFI = 0.046) was within the commonly accepted threshold of 0.01 (Bontempo and Hofer, 2006) supporting invariance. The scalar invariance model, which additionally constrains item intercepts to be equal across groups, did not degrade model fit relative to the metric model for either the 4- or 5-linking items model (S.Table 7). Thus, while we achieved partial invariance with 5-linking items, we found full measurement invariance with 4-linking items. Thus, we selected four-linking items, which still cover key aspects of cognitive enrichment (mainly reading a broad array of various materials and engaging in intellectually stimulating board games). Fig. 2A illustrates the data structure.

### 3.3. Statistical harmonization

The best fit in the reference study was provided by a CFA model that accounted for additional covariance among three reading items (i.e. reading newspapers, reading magazines, reading books) and between visiting museums and going to concerts (CFI = 0.962, TLI = 0.943, SRMR = 0.031).

**Fig. 3.**
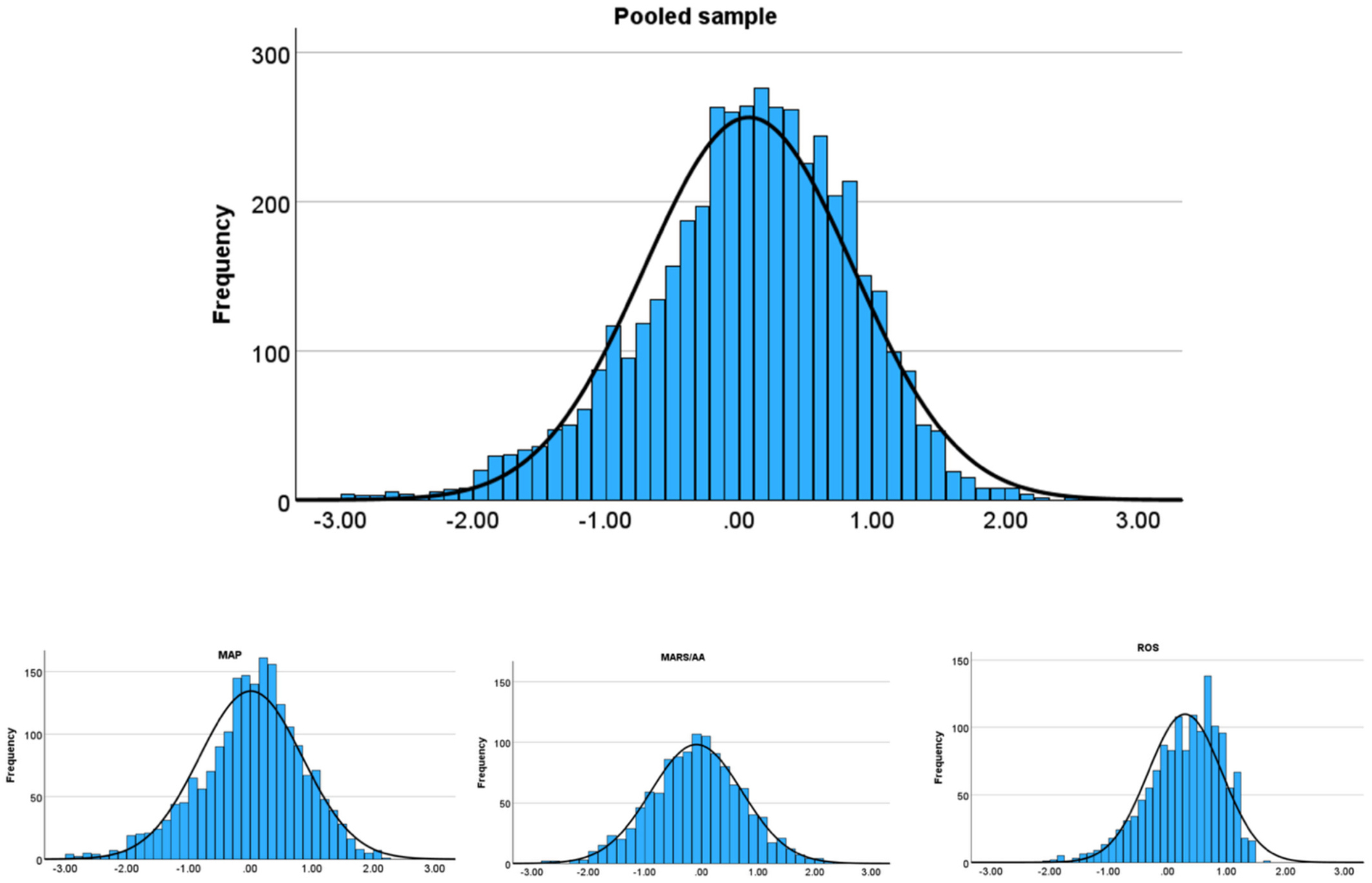
Distribution of harmonized factor score in the pooled sample and across the three studies. Histograms illustrating the distribution of the harmonized factor score for the pooled sample (n = 4550); Rush Memory and Aging Project (MAP, n = 1977), the Minority Aging Research Study (MARS) and Rush ADRD Clinical Core (n = 1191), and the Religious Orders Study (ROS, n = 1382). The histograms show a normal distribution for all studies except for a slight right skew for ROS.

We harmonized the pooled data via item banking (syntax in S.Files 1–4). Fig. 2B illustrates the co-calibration of the four studies. The factor scores were normally distributed in the pooled sample and in each study (Fig. 3). A higher score on the harmonized factor was highly correlated with each cohort-specific survey measure (r > 0.8, p < 0.001 in all cohorts) and with higher education (r > 0.3, p < 0.001 in all studies). Thus, results indicate that the harmonized score preserves cohort-specific ordering while improving comparability.

**Fig. 4.**
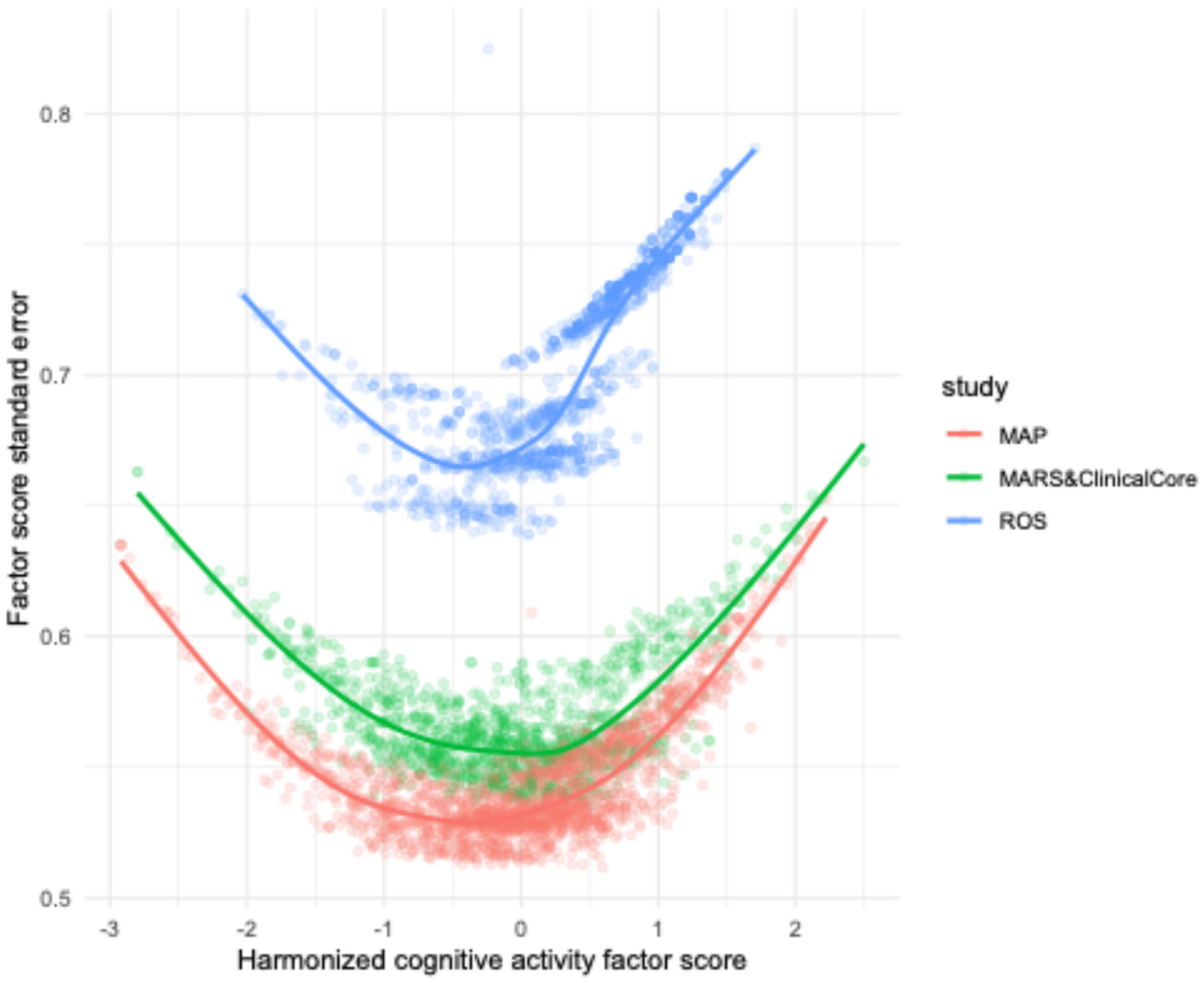
Factor score standard errors across the harmonized cognitive activity factor score stratified by study. Scatterplot with a smoothed line trend in bold, of the harmonized factor scores (x-axis) plotted against their standard errors, stratified by cohort (MAP in orange, MARS & Clinical Core in green, and ROS in blue). Standard errors increase at the extreme ends of the distribution, indicating (i) lower measurement precisions for very low (to the left) and very high (to the right) scores and (ii) cohort-specific differences in precision.

**Fig. 5.**
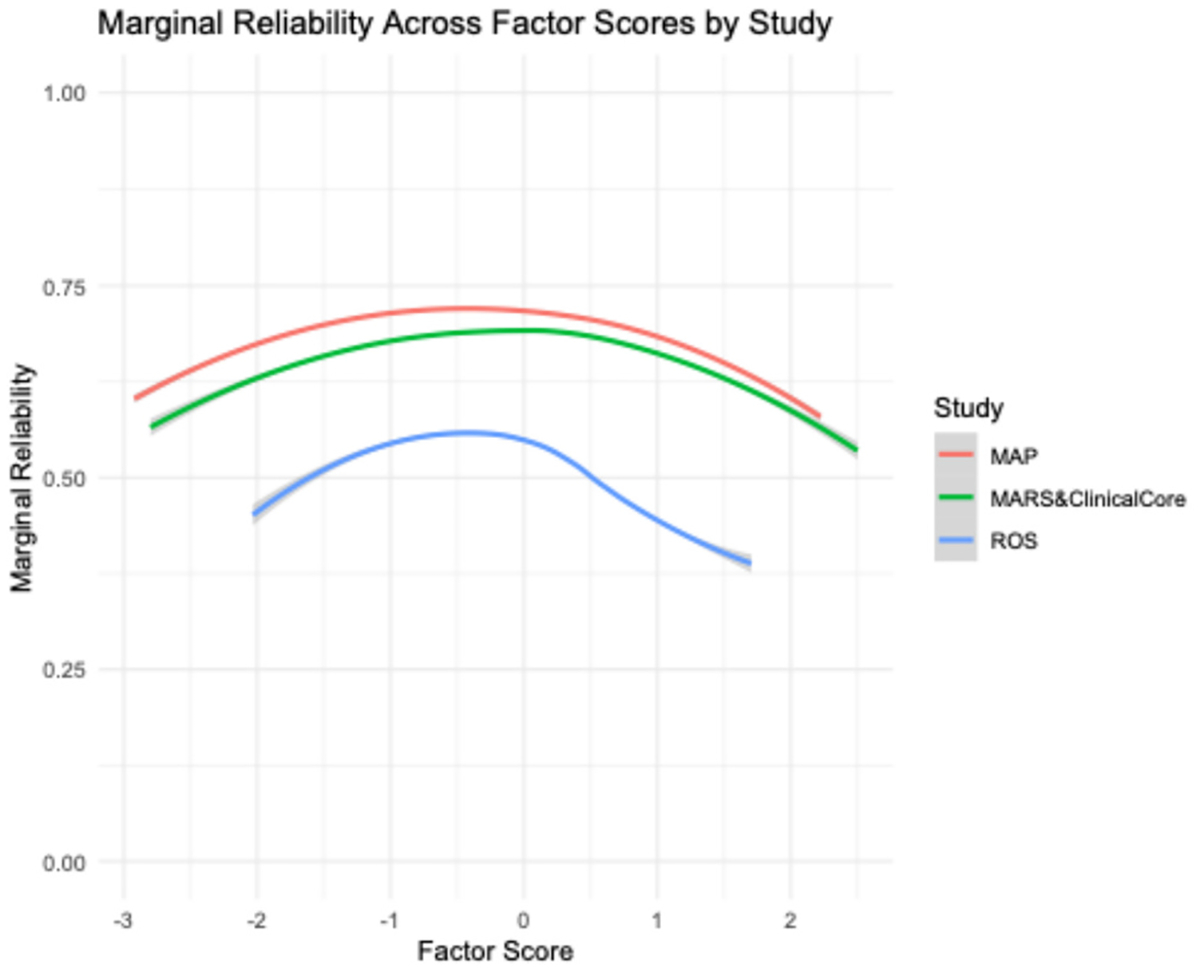
Marginal reliability of harmonized factor scores across the studies. Note. The Rush Memory and Aging Project (MAP) and the Minority Aging Research Study (MARS) and Clinical Core showed similar moderate marginal reliability; however, the Religious Orders Study (ROS) had relatively low reliability. Shaded gray is the standard error.

We illustrate the harmonized factor score and its standard errors stratified by sample in Fig. 4. The figure shows that measurement precision is highest between scores of −1 and 1, while standard errors increase at the extreme ends of the factor score, as expected. Notably, ROS exhibited lower precision compared with MAP, and MARS & Clinical Core, reflected by higher standard errors across the score range, especially at the extreme ends. Relatedly, marginal reliability, which in IRT can vary along the range of the latent trait reflecting cognitively stimulating activities, was higher for MAP, and MARS & Clinical Core than ROS (Fig. 5), reflecting fewer items (3 fewer items in ROS vs. 9 shared items in MAP, MARS & Clinical Core), and thus less information in the ROS sample. Reliability in MAP, and MARS & Clinical Core was sufficient for epidemiological research (Nunnally, 1978): r above 0.70 between scores of −1 and 1. Thus, caution is warranted when using ROS scores, particularly scores at the extreme ends, where both precision and reliability are relative to MAP, and MARS & Clinical Core.

### 3.4. External validation

We examined the validity of the harmonized factor as a measure of cognitive activity by estimating a series of models. First, we examined the association between the harmonized latent factor and incident AD dementia. During a mean follow-up of 8.2 years, 1158 participants developed AD dementia. More frequent participation in cognitively stimulating activities was associated with 20% lower hazards of developing AD dementia (HR = 0.80 per SD of the factor score, 95%CI = 0.74–0.86, p < 0.001). Similar magnitudes and directions of associations were observed in each cohort independently, albeit not significantly in MARS and Clinical Core given smaller sample sizes (Table 2). Relatedly, when we stratified by race we also found similar magnitudes and directions (862 events in Whites, HR = 0.77, 95%CI = 0.70, 0.84, p < 0.001 and 248 events in Blacks, HR = 0.85, 95%CI = 0.73, 0.99, p = 0.05).

**Fig. 6.**
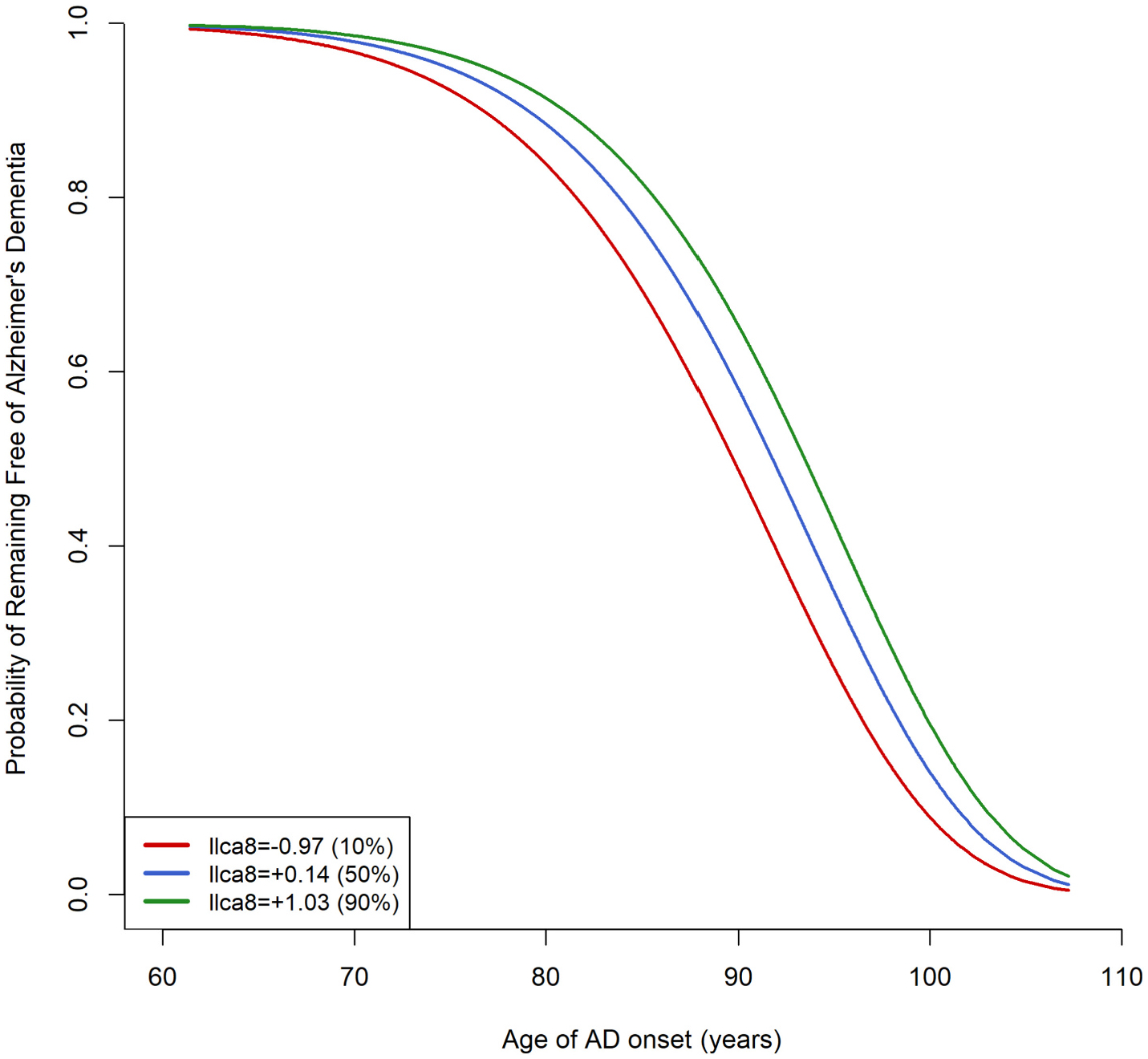
Probability of remaining dementia-free represented by percentiles of participations in late-life cognitively stimulating activities. Relation of low (10th percentile, in red), medium (50th percentile, in blue), and high (90th percentile, in green) engagement in late-life cognitively stimulating activities represented by the harmonized latent factor score, to probability of remaining dementia-free from Alzheimer's disease using an accelerated failure time model.

Higher level of participation in cognitively stimulating activities was associated with a later age of onset of AD dementia (est. = 0.02, 95%CI = 0.01, 0.03, p < 4 × 10^−8^). Participants with high engagement in cognitively stimulating activities (90th percentile) developed AD dementia at a mean age of 92.5 years relative to a mean age of onset of 88.7 years for participants with low engagement (10th percentile), a mean difference of almost 4 years. Fig. 6 shows the AD-free survival probabilities associated with different levels of participation in cognitively stimulating activities.

**Fig. 7.**
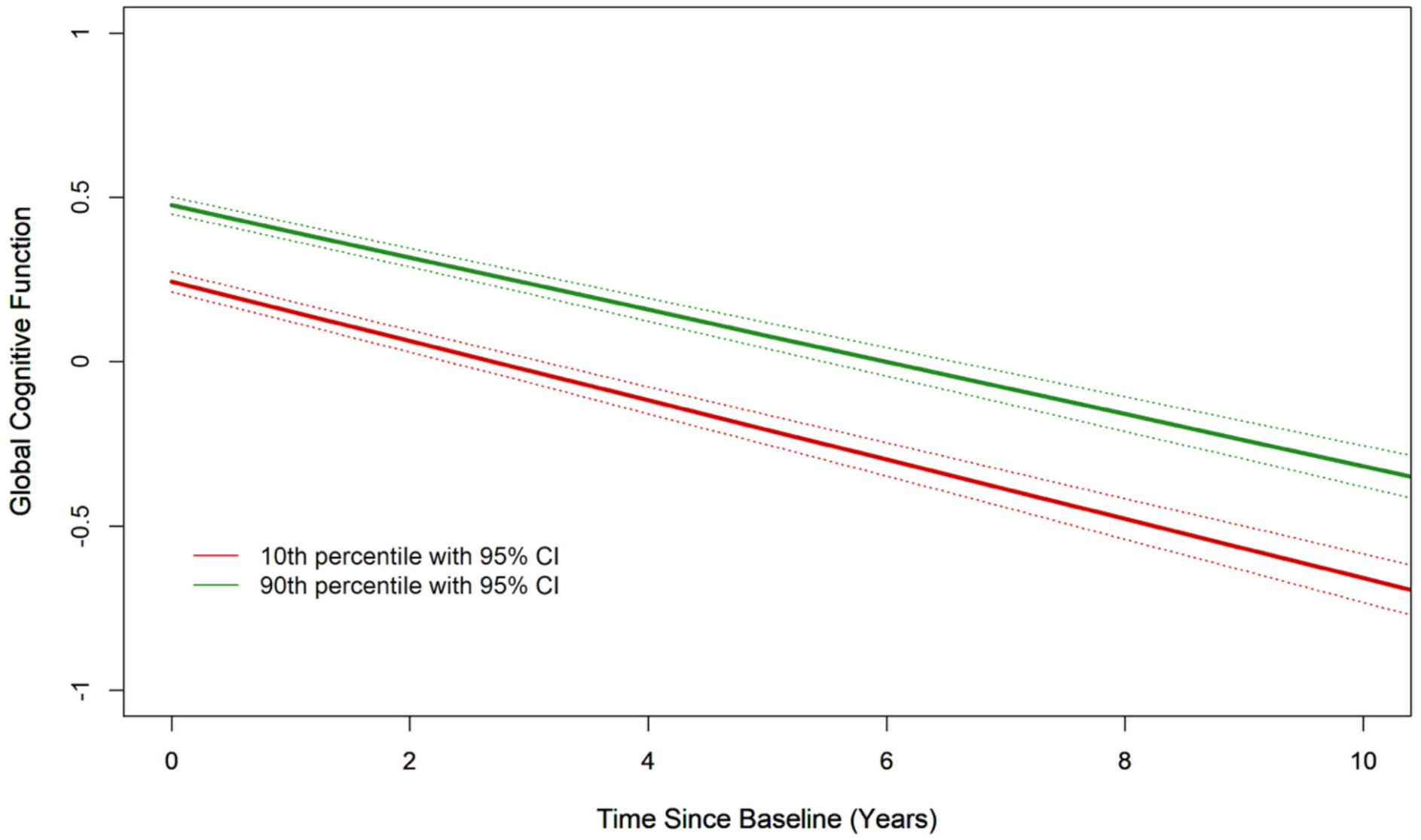
Association of participation in late-life cognitively stimulating activities to later-life cognitive decline from study baseline. Figure shows the predicted paths of decline in global cognition from baseline to 10 years of follow-up for individuals with high (90th percentile, green trajectory), and low (10th percentile, red trajectory) participation represented by a harmonized latent factor score, adjusting for age, sex, race, and education.

We next examined the association of the harmonized factor of cognitive stimulating activities with level and rate of decline in global cognition. After adjusting for age, sex, race, and education more frequent participation in cognitively stimulating activities was associated with higher level of cognitive function at baseline (est. = 0.116, S. E. = 0.01, p < 0.001), and with slower rate of cognitive decline (est. = 0.005, SE = 0.002, p = 0.02). To contextualize, an individual who is highly cognitively active (90th percentile) was estimated to decline at a 14% slower rate than a person who reports low participation in cognitively stimulating activities (10th percentile). For the person in the 90th percentile, this is equivalent to being 3 years younger than the person in the 10th percentile (Fig. 7).

In the individual studies the harmonized latent factor was associated with level of cognition across all studies, but only associated with rate of decline in MAP (Table 3). Results were similar when we stratified by race (Whites: level est. = 0.14, SE = 0.01, p < 0.001, slope est. =0.01, SE = 0.003, p = 0.01; Blacks: level est. = 0.04, SE = 0.02, p = 0.01; slope est. = 0.003, SE = 0.003, p = 0.0425).

In a subset of the sample who died and underwent autopsy (n = 2444; mean age at death = 91.4, SD = 6.2) we explored the frequencies of neuropathologies and examined the association of the harmonized factor to 9 neuropathologic indices, described above. Mean number of pathologies were 2.8 (range 0–8), with 120 (2%) participants having no pathologies, 282 (6%) having 1 pathology, 364 (8%) having 2 pathologies, and 946 (85%) having three or more. S. Fig. 2 shows an upset of the frequency and combination of pathologies stratified by participants with and without dementia at death. The upset shows that AD pathology was by far the most frequent pathology (n = 1174, 62%), though the majority of participants had a combination of co-occurring pathologies. We found no associations between any of the neuropathologic indices and the harmonized latent score, consistent with our previous findings (Wilson et al., 2013).

The presence of neuropathology degrades cognition. We thus tested whether the harmonized factor remained associated with cognition even after we regressed out the effects of pathology. Results showed that the effects of late-life cognitive activity on cognition remain significant even after we control for demographics and 9 common ADRD pathologic indices (level: est. = 0.149, SE = 0.04, p < 0.001; slope: est. = 0.011, SE = 0.004, p = 0.002) (Table 4). For the individual in the 90th percentile, this corresponded to being approximately 16 years younger than a person who reports low activity (10th percentile) after pathologies are accounted for.

When stratified by race, the association was found in White participants before (level:est. = 0.20, 0.03, p < 0.001; slope:est. = 0.01, SE = 0.002, p < 0.001) and after (level: est. = 0.167, SE = 0.02, p < 0.001, slope: est. = 0.010, SE = 0.003, p = 0.001) adjusting for pathologic indices, but not in Black participants (before:level est. = 0.06, SE = 0.05, p = 0.20, slope est. = 0.004, SE = 0.004,p = 0.33; after: level: est. = 0.20, SE = 0.17, p = 0.23, slope: est. = 0.022, SE = 0.02, p = 0.161), which is not surprising given the small N of 65 for Black participants.

## 4. Discussion

In this study we harmonized two measures of frequency of participation in cognitively stimulating activities across four cohort studies of older adults. By use of item-response theory we were able to make informed judgement during pre-statistical harmonization. The IRT model applied in this study provided us with the analytic methods needed to evaluate a set of items assessing frequency of participation in cognitively stimulating activities drawn from two separate surveys, to conduct formal tests of measurement invariance, and to create person-specific scale scores that are anchored to the same metric across data pooled from four cohort studies. Using these scores, we then tested for evidence of external validity by establishing associations of the harmonized latent factor with incident AD dementia, cognitive decline, and cognitive resilience.

In prior work we were limited to cohort-specific data analysis by studying the effects of frequency of participation in cognitively stimulating activities on ADRD cognitive outcomes (Wilson et al., 2003a; Wilson et al., 2007; Wilson et al., 2013; Wilson et al., 2002a; Wilson et al., 2003b; Barnes et al., 2006; Wilson et al., 2012; Marquine et al., 2012; Nsor et al., 2024) or by pooling scores across cohort studies that have the same measures i.e. analyzed as a collective whole (e.g. MAP and MARS) (Marquine et al., 2012; Lange-Maia et al., 2024). While these prior studies are fundamental in establishing associations across different populations, and in ensuring generalizability and replicability, the ability to pool data affords not only better power to detect associations (indeed, associations with cognitive decline were significant in the pooled sample but not in all studies individually) but also the ability to test novel hypotheses that could not be tested in an individual study. Most of the prior harmonization work has also mainly been restricted to harmonizing objective measures such as cognition (Gross et al., 2023), biomarker (Hohman, 2023), and brain imaging data (Pomponio et al., 2020). However, the harmonization of survey data that measures modifiable risk factors has not yet been adequately addressed.

Participation in leisure activities, such as cognitive and social activities, contribute to greater cognitive reserve over the life course (Song et al., 2022). By harmonizing this cognitive lifestyle measure, we combined psychosocial survey data collected across multiple cohort studies. All associations within the individual cohorts were consistently in the same direction, supporting construct validity. Further, effect sizes were generally larger and more statistically significant in the pooled sample than in most individual cohorts, reinforcing the value of harmonization. The harmonization enabled the equivalent evaluation of participation in cognitively stimulating activities across different populations, thus allowing parallel comparisons of important modifiable risk and resilience factors while maximizing the use of available data resources. Harmonizing and co-analyzing participant data across studies is central to achieve sufficient statistical power, refined subgroup analysis, enhanced generalizability, and enabling comparisons, cross-validation, and replication across datasets (Fortier et al., 2017).

The current projections on the modifiability of dementia via lifestyle factors (Livingston et al., 2024) necessitates the need to start aligning lifestyle surveys across studies and cultures. Inconsistent results in the associations between modifiable risk factors and ADRD outcomes across studies are often a reflection of sample size, different survey measures, heterogeneous populations, and a combination thereof. Existing large-scale cohort studies contain valuable information that can be integrated to test long-standing theories on reserve and resilience, and personality and cognitive health. Here, we had two measures that overlapped heavily, and which were administered to our participants across the four cohort studies by the same team of administrators, thus most of the groundwork was set. Harmonization initiatives provide opportunities to identify important associations and solutions to promote dementia prevention (Kivipelto et al., 2024). While there is enough evidence for reducing dementia-risk across the life-course, rigorous research is still warranted to comprehensively understand emerging risk factors, such as engagement in enriching leisure activities or past experiences, across heterogeneous populations. Data harmonization will be useful in obtaining additional compelling evidence on identification of novel modifiable risk factors, and effective and feasible preventive strategies (Kivipelto et al., 2024). Future work is warranted to determine guidelines on how to best combine survey data across large cohort studies.

Key strengths of our study include improved statistical power, greater heterogeneity in participant demographics, broader psychometric assessment of survey data, and maximizing the efficacy of time and money resources in the RADC gained from harmonizing data. Despite these strengths, the complexity of harmonizing measures across studies introduces important limitations. The harmonized survey data is based on self-reported cognitive activity, which may be subject to recall bias or social desirability bias. While an ideal scale that reflects participation in cognitively enriching activities does not exist, we have previously shown our scales have sound psychometric properties including internal consistency, re-test reliability, and external validity (Wilson et al., 2003b). Harmonization was based on a single measure taken at the baseline evaluation, as repeated measures were not available in ROS. Nonetheless, these scales have each previously demonstrated excellent predictive validity (Wilson et al., 2007). We treated all items in the CFA as reflective indicators of a latent construct; thus, we assumed all items reflect a latent trait encompassing cognitively stimulating activities. We acknowledge though that some items, e.g. watching television, listening to the radio, and visiting museums, may function more like formative or weak reflective indicators insofar as they contribute to defining the construct rather than simply reflect it and thus are not interchangeable. For instance, watching TV and visiting museums capture distinct behaviors, as opposed to items asking about reading books and reading magazines both of which involve similar activities and may be considered interchangeable. Therefore, the latent harmonized trait may be better understood as an aggregate of heterogeneous cognitively stimulating activities rather than a unidimensional trait. Reverse causality is also a concern as individuals with higher cognitive function may be more likely to engage in cognitive activities. To mitigate this bias, we excluded individuals with dementia at baseline. Finally, although self-reported survey-data as evaluated here captures multiple indicators of late-life cognitive activity, it may not fully reflect the cognitive demands or attentional requirements of those activities. Additionally, other sources of cognitively stimulating activities, e.g. drawing, knitting, or playing an instrument, are not captured by the harmonized score. That said, to our knowledge, this is the first study to harmonize valuable survey data that captures sources of individual differences in cognitive activity engagement across multiple cohorts. Future work is needed to build upon this effort by linking psychosocial measures across studies to comprehensively evaluate the impact of modifiable risk factors on later-life cognitive outcomes across heterogeneous populations.

## Supplementary Material

1

## Figures and Tables

**Table 1:** Characteristics of all study participants and stratified by study.

	Pooled sample	MAP	MARS & Clinical Core	ROS
Sample size, n	4550	1977	1191	1382
Age at baseline, mean years (SD)	76.9 (7.6)	79.8 (7.4)	73.1 (6.3)	75.5 (7.2)
Education, years (SD)	16.0 (3.7)	15.0 (3.4)	14.8 (3.2)	18.5 (3.4)
Female, n (%)	3407 (75)	1479 (75)	940 (78)	988 (71)
Late life cognitive activities survey in MAP and MARS & Clinical Core (range: 1–5)	–	3.2 (0.6)	2.9 (0.6)	–
Late life cognitive activities survey in ROS (range: 1–5)	–	–	–	3.5 (0.5)

*Note*. MAP = Rush Memory and Aging Project. MARS = Minority Aging Research Study. ROS = Religious Orders Study.

**Table 2:** Hazardss of incident Alzheimer's disease (AD) dementia associated with late-life engagement in cognitive activities in the pooled sample and stratified by study. N incidence in pooled sample = 1158; MAP = 493, MARS & Clinical Core = 188, ROS = 477.

		All	MAP	MARS & Clinical Core	ROS
	Model term	HR (95%CI) p	HR (95%CI) p	HR (95%CI) p	HR (95%CI) p
Incident AD dementia	Harmonized latent factor	0.80 (0.74, 0.86), <0.001	0.76 (0.68, 0.84), <0.001	0.88 (0.74,1.06), 0.188	0.81 (0.70, 0.94), 0.006
	9-item survey in MAP and MARS & Clinical Core	–	0.67 (0.58, 0.77), <0.001	0.81 (0.63, 1.04), 0.0983	–
	7-item survey in ROS	–	–	–	0.77 (0.66, 0.91), 0.002

**Note.** HR = hazard ratio. MAP = Memory and Aging Project. MARS = Minority Aging Research Study. ROS = Religious Orders Study. Each estimate is a separate proportional hazards model that has been adjusted for age at baseline, sex, and education. In All, MAP, and ROS, models were further adjusted for race.

**Table 3:** Association of the harmonized latent factor representing late-life participation in cognitive activities to cognitive level and slope in the pooled sample and stratified by study.

Model term	All (n = 4550)	MAP	MARS & Clinical Core	ROS
	Estimate (SE) p	Estimate (SE) p	Estimate (SE) p	Estimate (SE) p
Time (from baseline)	−0.081 (0.002) <0.001	−0.097 (0.003), <0.001	−0.046 (0.003), <0.001	−0.084 (0.004), <0.001
Harmonized latent factor	0.121 (0.01), <0.001	0.15 (0.02) <0.001	0.064 (0.018), <0.001	0.098 (0.021) <0.001
Time X Harmonized latent factor	0.005 (0.002), 0.02	0.01 (0.004) 0.003	−0.001 (0.003), 0.761	0.003 (0.005), 0.506
**Individual surveys**				
9-item survey	–	0.239 (0.02) <0.001	0.114 (0.02), <0.001	–
Time X 9-item survey	–	0.014 (0.005) 0.002	0.0004 (0.005), 0.932	–
7-item survey	–	–	–	0.101 (0.02) <0.001
Time X 7-item survey	–	–	–	0.0090 (0.01), 0.09

*Note*. MAP = Rush Memory and Aging Project. MARS = Minority Aging Research Study. ROS = Religious Orders Study. SE = standard error. Each column is a linear-mixed effects model that controlled for age at baseline, sex, and education. In the pooled sample (All), MAP, and ROS, models were further adj0.14444usted for race.

**Table 4:** Association of frequency in late life cognitive activities to cognitive level and slope after adjustment for AD/ADRD neuropathologic burden in the pooled sample and stratified by study (n = 2444).

Model term	All	MAP	MARS & Clinical Core	ROS
	Estimate (SE), p	Estimate (SE), p	Estimate (SE), p	Estimate (SE), p
Time before death	0.008 (0.008), 0.300	−0.0002 (0.015), 0.991	−0.064 (0.006) <0.001	0.0151 (0.0102), 0.1396
Harmonized factor score	0.149 (0.04), <0.001	0.179 (0.048), <0.001	−0.031 (0.068), 0.660	0.068 (0.06), 0.328
Time before death X harmonized factor score	0.011 (0.004), 0.002	0.014 (0.006), 0.012	−0.007 (0.006), 0.253	0.005 (0.005), 0.344
**Individual Surveys**				
Nine-item survey	NA	0.288 (0.06), <0.001	0.025 (0.09), 0.80	
Time before death X 9-item survey	NA	0.021 (0.008), 0.006	−0.005 (0.009), 0.570	
7-item survey	NA	NA		0.100 (0.07), 0.15
Time before death X 7-item survey	NA	NA		0.008 (0.01), 0.156

**Note.** MAP = Rush Memory and Aging Project. MARS = Minority Aging Research Study. ROS = Religious Orders Study. Each column is a linear-mixed effects model that controlled for age at death, sex, education, race, and 9 common Alzheimer's disease and related dementia pathologic indices. In the pooled sample (All), MAP, and ROS, models were further adjusted for race.

## Data Availability

Data will be made available on request.

## Appendix A. Supplementary data

Supplementary data to this article can be found online at https://doi.org/10.1016/j.exger.2026.113069.
