# Supplementary material for: Harmonization of late-life participation in cognitively stimulating activities across four cohort studies of cognitive aging
Source: Exp Gerontol. Author manuscript; Available in PMC 2026 Jun 11. (PMC13255081; doi:10.1016/j.exger.2026.113069)
Supplement: 1 [file NIHMS2178265-supplement-1.docx]

**Supplementary Material**

**S. Table 1.** **Illustration of the data structure. Highlighted in green are the overlapping items, and in gray, the non-overlapping items.**

|  | **MAP/MARS&Clinical Core** | **ROS** | **Scale** | **Comparability** |
| --- | --- | --- | --- | --- |
| 1 | Thinking of the last year, how often do you read newspapers? | How often do you read newspapers? | Would you say every day or almost every day, several times a week, several times a month, several times a year or once a year or less? | High |
| 2 | During the past year, how often did you read magazines? | How often do you read magazines? | Would you say every day or almost every day, several times a week, several times a month, several times a year or once a year or less? | High |
| 3 | During the past year, how often did you read books? | How often do you read books? | Would you say every day or almost every day, several times a week, several times a month, several times a year or once a year or less? | High |
| 4 | During the past year, how often did you play games like checkers or other board games, cards, puzzles, etc? | How often do you play games such as cards, checkers, crosswords, or other puzzles or games? | Would you say every day or almost every day = 5, several times a week = 4, several times a month = 3, several times a year = 2 or once a year or less =1? | High |
| 5 | In the last ten years, how many times did you visit a museum? | How often do you go to museums? | MAP/MARS&Clinical Core Never (1), 1-2 times (2), 3-9 times (3), 10-19 times (4), more than 20 times (5).  ROS: 5= Every day or almost every day 4= Several times a week 3= Several times a month 2= Several times a year 1= Once a year or less | Medium |
| 6 | About how much time do you spend reading each day? |  | None = 1, less than one hour =2, one to less than two hours =3, two to less than three hours =4, three or more hours =5. | N/A |
| 7 | In the last ten years, how often did you visit a library? |  | Would you say every day or almost every day, several times a week, several times a month, several times a year or once a year or less? | N/A |
| 8 | During the past year, how often did you write letters? |  | Would you say every day or almost every day, several times a week, several times a month, several times a year or once a year or less? | N/A |
| 9 | In the last ten years, how many times did you attend a concert, play, or musical? |  | Never, 1-2 times, 3-9 times, 10-19 times, more than 20 times. | N/A |
| 10 |  | How often do you watch television? | Would you say every day or almost every day, several times a week, several times a month, several times a year or once a year or less? | N/A |
| 11 |  | How often do you listen to the radio? | Would you say every day or almost every day, several times a week, several times a month, several times a year or once a year or less? | N/A |

**Note.** Shaded green indicates linking items, shaded orange and gray indicate non-linking items. Even though some cohorts shared items, we only fixed parameters in the models for the green shaded linking items. Comparability of items was done during the pre-statisticial harmonization process and indicates comparability in the stem question prompt and the response options. AZ rated each item as a confident linking item (i.e. high comparability), a tentative linking item (i.e. medium comparability), and a non-linking item (i.e. unique to one or some cohorts but not to others). Comparability was then formally tested by examining factor loadings of these items within each cohort using confirmatory factor analysis and differential item functioning.

**S. Table 2. Pearson correlation and p-value amongst items from the late-life cognitive activity survey in the Rush Memory and Aging Project (N=1977).**

| MAP | Magazines | Read books | Play games | Visit museum | Time reading | Visit library | Write letters | Attend concerts |
| --- | --- | --- | --- | --- | --- | --- | --- | --- |
| Newspapers | 0.41 (<0.001) | 0.13 (<0.001) | 0.09 (<0.001) | 0.18 (<0.001) | 0.13 (<0.001) | 0.16 (<0.001) | 0.24 (<0.001) | 0.18 (<0.001) |
| Magazines | - | 0.17 (<0.001) | 0.03 (0.16) | 0.20 (<0.001) | 0.12 (<0.001) | 0.18 (<0.001) | 0.23 (<0.001) | 0.16 (0.001) |
| Read books |  | - | 0.05 (0.04) | 0.20 (<0.001) | 0.31 (<0.001) | 0.16 (<0.001) | 0.43 (<0.001) | 0.18 (<0.001) |
| Play games |  |  | - | 0.11 (<0.001) | 0.10 (<0.001) | 0.09 (<0.001) | 0.01 (0.55) | 0.15 (<0.001) |
| Visit museums |  |  |  | - | 0.22 (<0.001) | 0.13 (<0.001) | 0.22 (<0.001) | 0.55 (<0.001) |
| Time reading |  |  |  |  | - | 0.17 (<0.001) | 0.26 (<0.001) | 0.16 (<0.001) |
| Visit library |  |  |  |  |  | - | 0.16 (<0.001) | 0.14 (<0.001) |
| Write letters |  |  |  |  |  |  | - | 0.19 (<0.001) |

**S. Table 3. Pearson correlation and p-value amongst items from the late-life cognitive activity survey in Minority Aging Research Study and Rush Alzheimer’s Disease Research Center (ADRC) Clinical Core (N=1191).**

| MARS & Clinical Core | Magazines | Read books | Play games | Visit museum | Time reading | Visit library | Write letters | Attend concerts |
| --- | --- | --- | --- | --- | --- | --- | --- | --- |
| Newspapers | 0.40 (<0.001) | 0.07 (0.015) | -0.02 (0.613) | 0.13 (<0.001) | 0.12 (<0.001) | 0.09 (0.002) | 0.142 (<0.001) | 0.144 (<0.001) |
| Magazines | - | 0.23 (<0.001) | 0.09 (0.008) | 0.20 (<0.001) | 0.14 (<0.001) | 0.20 (<0.001) | 0.22 (<0.001) | 0.20 (0.001) |
| Read books |  | - | 0.12 (<0.001) | 0.16 (<0.001) | 0.23 (<0.001) | 0.20 (<0.001) | 0.41 (<0.001) | 0.07 (<0.001) |
| Play games |  |  | - | 0.04 (0.23) | 0.10 (<0.001) | 0.02 (0.58) | 0.12 (<0.001) | 0.05 (0.136) |
| Visit museums |  |  |  | - | 0.29 (<0.001) | 0.18 (<0.001) | 0.16 (<0.001) | 0.51 (<0.001) |
| Time reading |  |  |  |  | - | 0.17 (<0.001) | 0.16 (<0.001) | 0.14 (<0.001) |
| Visit library |  |  |  |  |  | - | 0.14 (<0.001) | 0.14 (<0.001) |
| Write letters |  |  |  |  |  |  | - | 0.19 (<0.001) |

**S. Table 4. Pearson correlation and p-value amongst items from the late-life cognitive activity survey in Religious Orders Study (N=1382).**

| ROS | Magazines | Read books | Play games | Visit museums | Watch television | Listen radio |
| --- | --- | --- | --- | --- | --- | --- |
| Newspapers | 0.29 (<0.001) | 0.09 (<0.001) | 0.02 (0.468) | 0.06 (0.02) | 0.07 (0.02) | 0.06 (0.02) |
| Magazines | - | 0.21 (<0.001) | 0.02 (0.577) | 0.09 (<0.001) | 0.18 (0.516) | 0.09 (<0.001) |
| Read books |  | - | 0.07 (0.01) | 0.07 (0.006) | -0.02 (0.374) | 0.100 (<0.001) |
| Play games |  |  | - | 0.04 (0.07) | 0.10 (<0.001) | 0.05 (0.06) |
| Visit museums |  |  |  | - | 0.07 (0.009) | 0.15 (<0.001) |
| Listen radio |  |  |  |  | - | 0.11 (<0.001) |

**S. Table 5. Mean and standard deviation (SD) of items by cohort.**

| **Items** | **MAP** | **MARS &Clinical Core** | **ROS** |
| --- | --- | --- | --- |
| *How often do you read newspapers? | 4.2 (1.3) | 3.9 (1.4) | 4.4 (1.1) |
| *How often did you read magazines? | 3.4 (1.2) | 3.6 (1.2) | 3.5 (1.1) |
| *How often did you read books? | 3.4 (1.4) | 3.2 (1.4) | 4.2 (1.3) |
| *How often did you play games like checkers or other board games, cards, puzzles, etc? | 3.0 (1.4) | 3.1 (1.5) | 3.1 (1.5) |
| *How many times did you visit a museum (in the last 10 years for MAP/MARS/Clinical Core)? | 3.1 (1.3) | 2.8 (1.2) | 1.3 (0.5) |
| About how much time do you spend reading each day? | 2.4 (1.1) | 1.9 (1.0) | - |
| In the last ten years, how often did you visit a library? | 2.3 (1.1) | 1.7 (1.0) | - |
| During the past year, how often did you write letters? | 3.3 (1.0) | 3.0 (1.0) | - |
| In the last ten years, how many times did you attend a concert, play, or musical? | 3.8 (1.2) | 3.5 (1.2) | - |
| How often do you watch television? | - | - | 4.5 (1.0) |
| How often do you listen to the radio? | - | - | 3.8 (1.6) |

**S. Table 6.** **Standardized factor loadings of all items in each cohort.** Step A2 in the pre-harmonization process: Run confirmatory factor analysis in each study.

| **Items** | **MAP** | **MARS &Clinical Core** | **ROS** |
| --- | --- | --- | --- |
| *How often do you read newspapers? | 0.48 | 0.33 | 0.22 |
| *How often did you read magazines? | 0.33 | 0.41 | 0.20 |
| *How often did you read books? | 0.48 | 0.68 | 0.33 |
| *How often did you play games like checkers or other board games, cards, puzzles, etc? | 0.24 | 0.14 | 0.22 |
| *How many times did you visit a museum? | 0.56 | 0.13 | 0.39 |
| About how much time do you spend reading each day? | 0.38 | 0.47 | - |
| In the last ten years, how often did you visit a library? | 0.41 | 0.48 | - |
| During the past year, how often did you write letters? | 0.47 | 0.39 | - |
| In the last ten years, how many times did you attend a concert, play, or musical? | 0.47 | 0.73 | - |
| How often do you watch television? | - | - | 0.31 |
| How often do you listen to the radio? | - | - | 0.36 |

Asterisk* denotes anchor item.

**S. Table 7. Measurement invariance indices for the five- and four- item models.** Steps A3 and A4 in the pre-harmonization process: Identify linking items and test for measurement invariance for five and four linking items across all three cohorts.

|  | **Configural** | **Metric** | **Scalar** |
| --- | --- | --- | --- |
| Five items: newspaper, magazine, books, games, museum | CFI=0.930  TLI=0.860  RMSEA=0.060  SRMR=0.030 | CFI=0.884  TLI=0.849  RMSEA=0.062  SRMR=0.043 | CFI=0.884  TLI=0.849  RMSEA=0.062  SRMR=0.043 |
| Four items: newspaper, magazine, books, games (no museum) | CFI=0.967  TLI=0.929  RMSEA=0.049  SRMR=0.020 | CFI=0.964  TLI=0.946  RMSEA=0.042  SRMR=0.026 | CFI=0.964  TLI=0.946  RMSEA=0.042  SRMR=0.026 |

***
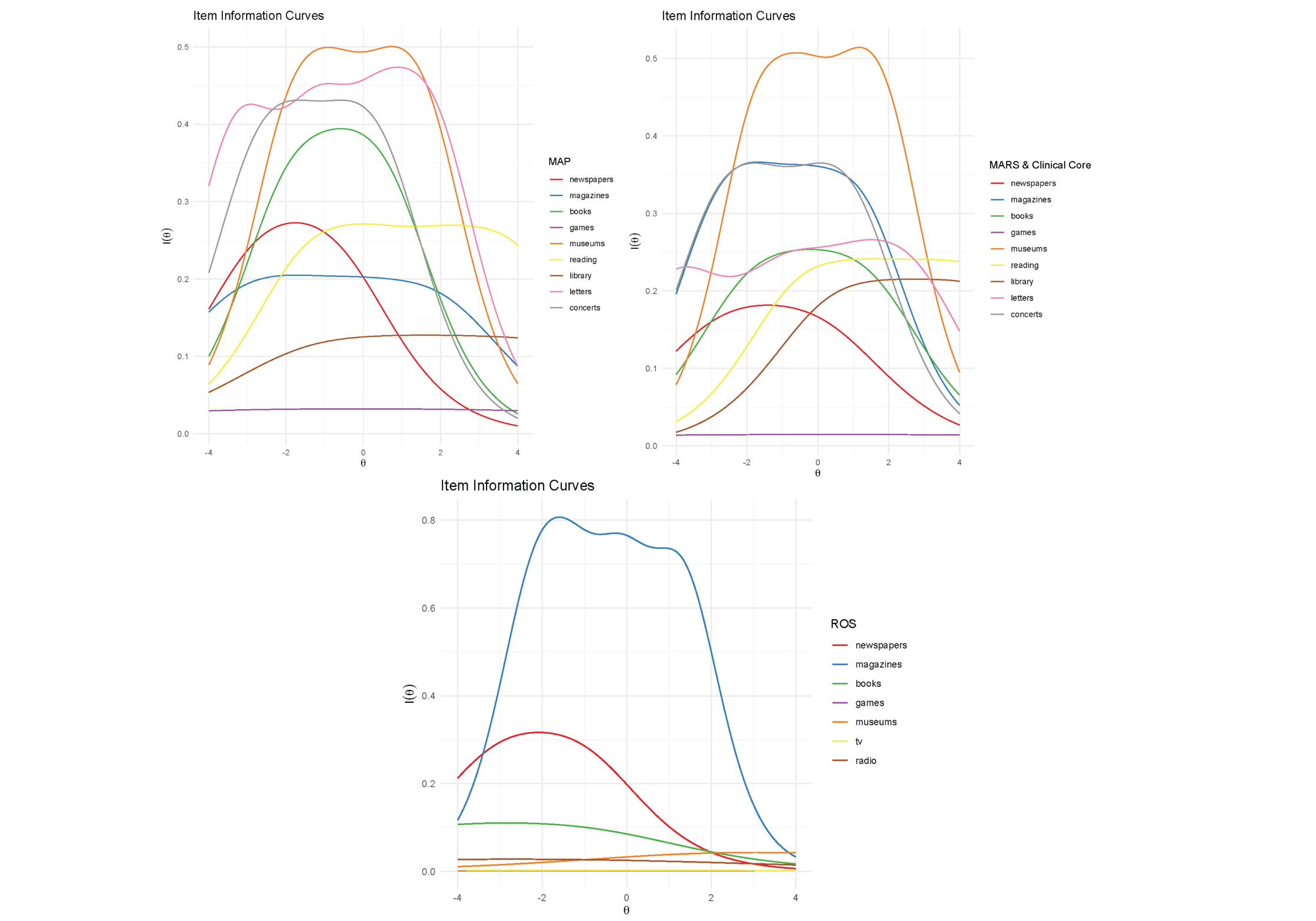
*Supplementary Figures**

**S. Figure 1. Item Information Curves for each study.** The plot illustrates the information provided by the items across the range of theta (x-axis). The peak of the curves shows where the cognitive activity items yield the greatest information about respondents (in the z-score range -4 to 4). A higher curve indicates the item provides more information, while flatter curves indicate low discrimination. As can be seen three of the anchor items were a good discriminator of cognitive activity engagement, i.e. the probability of a higher response to these items (representing higher cognitive engagement) would increase more rapidly as the level of the engagement increases on the item while item 4 (playing games) does not provide as much discriminating information on the latent trait on engagement in cognitively stimulating activities as much as items 1-3 which are more directly related to the trait. Nevertheless, the games item was included as a linking indicator because it was conceptually similar across studies.


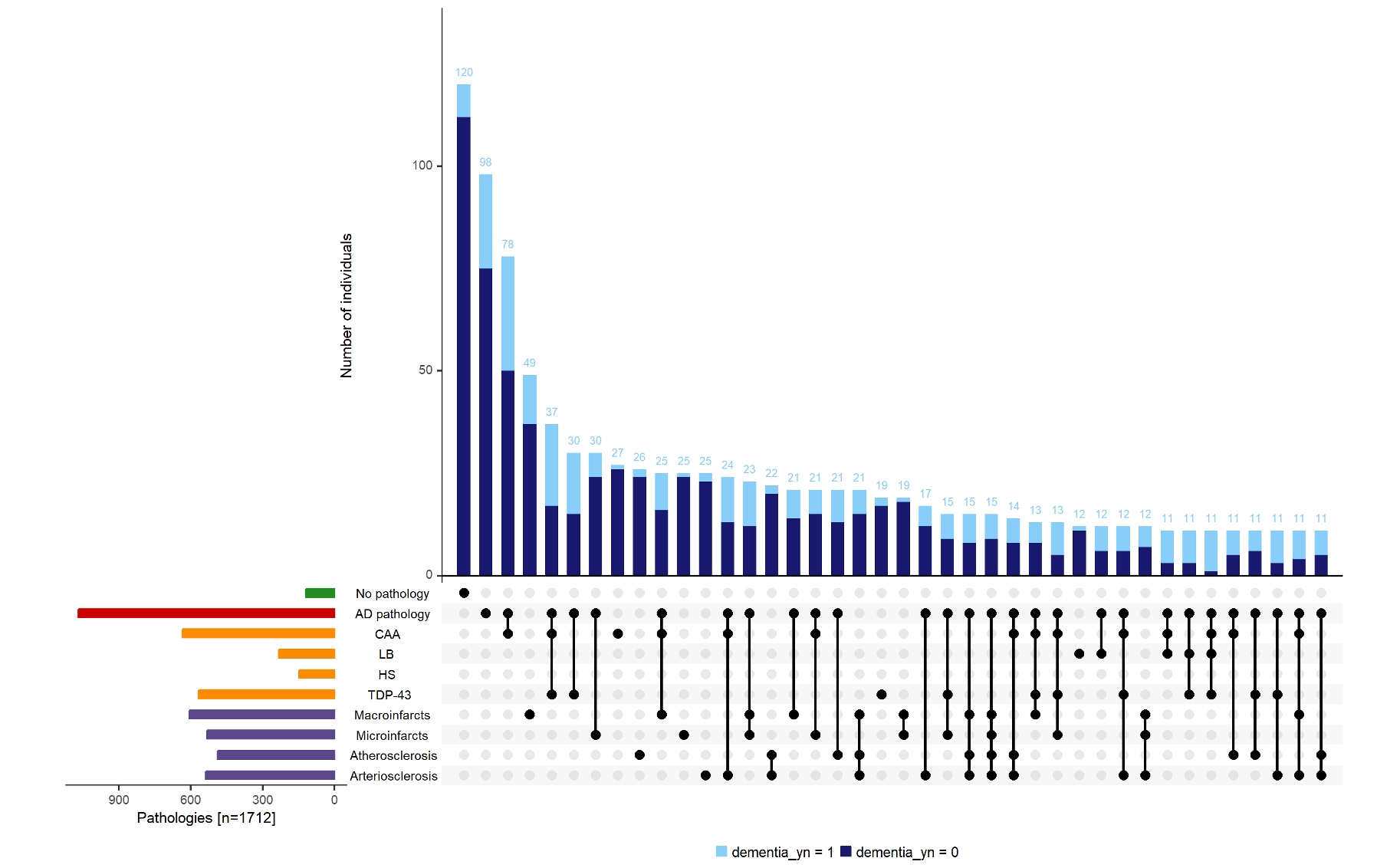


**S. Figure 2. Upset of burden of brain pathologies in pooled sample of participants.**

Histograms in the main panel show the frequencies of the neuropathologic indices for all participants stratified by presence of dementia at death. The bar chart shows the frequencies of each brain pathology in the pooled sample. The connected black dots indicate the specific combination of the neuropathologies represented. As illustrated, while AD pathology is the most common brain pathology, most brain neuropathologic indices frequently co-occur.

**Supplementary files providing syntax for harmonization, outlining Step B from Figure 1.**

**S. File 1. Step B1 and 2: B1) Estimate item parameters in reference study, and B2) save parameters into item-bank.**

**MPlus syntax for item-banking approach by running confirmatory factor analysis using the Rush Memory and Aging Project cohort study as reference and determining item parameters of the model.**

TITLE: confirmatory FA in MAP - all items all cohorts;

DATA: FILE is Macintosh HD//Users/azammit/Documents/cogact/itemlevel.csv;

NOBSERVATIONS = 4551;

VARIABLE: NAMES ARE

projid study

! MAP = 1

! ROS = 2

! MARS & Clinical Core = 3

agebl msex educ cohort

cogactrmma

!anhors

anchor1 anchor2 anchor3

!games museum

gamesmap museummap

gamesmars museummars

gamesros museumros

!ros

tvros radioros

!map

readamap libmap lettermap concertmap

!marsaa

readamarsaa libmarsaa lettermarsaa concertmarsaa;

USEVARIABLES = anchor1 anchor2 anchor3

gamesmap museummap

readamap libmap lettermap concertmap;

CATEGORICAL ARE anchor1 anchor2 anchor3

gamesmap museummap

readamap libmap lettermap concertmap;

USEOBSERVATIONS ARE study ==1;

MISSING = ALL(999);

IDVARIABLE = projid;

ANALYSIS:

estimator=wlsmv ;

! processors=8;

PARAMETERIZATION=THETA ;

OUTPUT:

svalues;

stdyx;

sampstat;

modindices(all);

mod(10);

MODEL: CogAct1 BY anchor1* anchor2 anchor3

gamesmap museummap

readamap libmap lettermap concertmap;

CogAct1@1;

[CogAct1@0];

anchor1 with anchor2;

anchor1 with anchor3;

museummap with concertmap;

**Step B3-5: B3) Constraining linking items, B4) freely estimating new item parameters, B5) save parameters into item bank.**

**S. File 2. Step 2: Confirmatory factor analysis in MARS & Rush ADRC clinical core to generate s-values for non-linking items –**

TITLE: confirmatory FA in MARS & clinical core - all items all cohorts for harmoniation;

DATA: FILE is Macintosh HD//Users/azammit/Documents/cogact/itemlevel.csv;

NOBSERVATIONS = 4551;

VARIABLE: NAMES ARE

projid study

! MAP = 1

! ROS = 2

! MARS/Clinical Core = 3

agebl msex educ cohort

cogactrmma

!anhors

anchor1 anchor2 anchor3

!games museum

gamesmap museummap

gamesmars museummars

gamesros museumros

!ros

tvros radioros

!map

readamap libmap lettermap concertmap

!marsaa

readamarsaa libmarsaa lettermarsaa concertmarsaa;

USEVARIABLES = anchor1 anchor2 anchor3

gamesmars museummars

readamarsaa libmarsaa lettermarsaa concertmarsaa

;

CATEGORICAL ARE anchor1 anchor2 anchor3

gamesmars museummars

readamarsaa

libmarsaa lettermarsaa concertmarsaa

;

USEOBSERVATIONS ARE study ==3;

MISSING = ALL(999);

IDVARIABLE = projid;

ANALYSIS:

estimator=wlsmv ;

! processors=8;

PARAMETERIZATION=THETA ;

OUTPUT:

svalues;

stdyx;

sampstat;

modindices(all);

mod(10);

MODEL:

cogact1 BY anchor1@0.55347;

cogact1 BY anchor2@0.40020;

cogact1 BY anchor3@0.90272;

cogact1 BY gamesmars@0.17480 ;

cogact1 BY museummars* ;

cogact1 BY readamarsaa* ;

cogact1 BY libmarsaa* ;

cogact1 BY lettermarsaa* ;

cogact1 BY concertmarsaa*

;

Cogact1@1;

[Cogact1@0];

[ anchor1$1@-1.49724 ];

[ anchor1$2@-1.29566 ];

[ anchor1$3@-0.88050 ];

[ anchor1$4@-0.43252 ];

[ anchor2$1@-1.47788 ];

[ anchor2$2@-0.95048 ];

[ anchor2$3@-0.04921 ];

[ anchor2$4@0.90495 ];

[ anchor3$1@-1.42442 ];

[ anchor3$2@-0.70078 ];

[ anchor3$3@-0.14289 ];

[ anchor3$4@0.39413 ];

[ gamesmars$1@-0.84597 ];

[ gamesmars$2@-0.34027 ];

[ gamesmars$3@0.24233 ];

[ gamesmars$4@0.87548 ];

anchor1 WITH anchor2@0.36473;

anchor1 WITH anchor3@-0.23853;

museummars WITH concertmarsaa;

**S. File 3. Step 3:** Confirmatory factor analysis in ROS to generate s-values for non-linking items - ;

TITLE: confirmatory FA in ROS - all items all cohorts; fixing anchor items and freely estimating nonanchor items

DATA: FILE is Macintosh HD//Users/azammit/Documents/cogact/itemlevel.csv;

NOBSERVATIONS = 4551;

VARIABLE: NAMES ARE

projid study

! MAP = 1

! ROS = 2

! MARS/Clinical Core = 3

agebl msex educ cohort

cogactrmma

!anhors

anchor1 anchor2 anchor3

!games museum

gamesmap museummap

gamesmars museummars

gamesros museumros

!ros

tvros radioros

!map

readamap libmap lettermap concertmap

!marsaa

readamarsaa libmarsaa lettermarsaa concertmarsaa;

USEVARIABLES = anchor1 anchor2 anchor3

gamesros museumros

tvros radioros;

CATEGORICAL ARE anchor1 anchor2 anchor3

gamesros museumros

tvros radioros

;

USEOBSERVATIONS ARE study ==2;

MISSING = ALL(999);

IDVARIABLE = projid;

ANALYSIS:

estimator=wlsmv ;

! processors=8;

PARAMETERIZATION=THETA ;

OUTPUT:

svalues;

stdyx;

sampstat;

modindices(all);

mod(10);

MODEL:

cogact1 BY anchor1@0.55347;

cogact1 BY anchor2@0.40020;

cogact1 BY anchor3@0.90272;

cogact1 BY gamesros@0.17480;

cogact1 BY museumros*;

cogact1 BY tvros* ;

cogact1 BY radioros* ;

Cogact1@1;

[Cogact1@0];

[ anchor1$1@-1.49724 ];

[ anchor1$2@-1.29566 ];

[ anchor1$3@-0.88050 ];

[ anchor1$4@-0.43252 ];

[ anchor2$1@-1.47788 ];

[ anchor2$2@-0.95048 ];

[ anchor2$3@-0.04921 ];

[ anchor2$4@0.90495 ];

[ anchor3$1@-1.42442 ];

[ anchor3$2@-0.70078 ];

[ anchor3$3@-0.14289 ];

[ anchor3$4@0.39413 ];

[ gamesros$1@-0.84597 ];

[ gamesros$2@-0.34027 ];

[ gamesros$3@0.24233 ];

[ gamesros$4@0.87548 ];

anchor1 WITH anchor2@0.36473;

anchor1 WITH anchor3@-0.23853;

**S. File 4. MPlus syntax running confirmatory factor analysis in the pooled data where linking items parameters are fixed, and factor scores are generated.**

TITLE: harmonization8 using four anchor items and all items all cohorts;

DATA: FILE is Macintosh HD//Users/azammit/Documents/cogact/itemlevel.csv;

NOBSERVATIONS = 4551;

VARIABLE: NAMES ARE

projid study

! MAP = 1

! ROS = 2

! MARS/Clinical Core = 3

agebl msex educ cohort

cogactrmma

!anhors

anchor1 anchor2 anchor3

!games museum

gamesmap museummap

gamesmars museummars

gamesros museumros

!ros

tvros radioros

!map

readamap libmap lettermap concertmap

!marsaa

readamarsaa libmarsaa lettermarsaa concertmarsaa;

USEVARIABLES = anchor1 anchor2 anchor3

gamesmap museummap

gamesmars museummars

gamesros museumros

tvros radioros

readamap libmap lettermap concertmap

readamarsaa libmarsaa lettermarsaa concertmarsaa;

CATEGORICAL ARE anchor1 anchor2 anchor3

gamesmap museummap

gamesmars museummars

gamesros museumros

tvros radioros

readamap libmap lettermap concertmap

readamarsaa libmarsaa lettermarsaa

concertmarsaa;

MISSING = ALL(999);

IDVARIABLE = projid;

auxiliary = study;

ANALYSIS:

estimator=MLR ;

LINK=PROBIT;

OUTPUT:

stdyx;

SAVEDATA:

FILE= RMMA_harmonization8.dat;

SAVE= FSCORES;

MODEL:

cogact1 BY anchor1@0.55347;

cogact1 BY anchor2@0.40020;

cogact1 BY anchor3@0.90272;

cogact1 BY gamesmap@0.17480;

cogact1 BY museummap@0.47747;

cogact1 BY readamap@0.55953;

cogact1 BY libmap@0.38859;

cogact1 BY lettermap@0.86398;

cogact1 BY concertmap@0.41811;

cogact1 BY gamesmars@0.17480;

cogact1 BY museummars@0.45424;

cogact1 BY readamarsaa@0.51966;

cogact1 BY libmarsaa@0.45878;

cogact1 BY lettermarsaa@0.69006;

cogact1 BY concertmarsaa@0.33157;

cogact1 BY gamesros@0.17480;

cogact1 BY museumros@0.29576;

cogact1 BY tvros@0.06459;

cogact1 BY radioros@0.25315;

[ anchor1$1@-1.49724 ];

[ anchor1$2@-1.29566 ];

[ anchor1$3@-0.88050 ];

[ anchor1$4@-0.43252 ];

[ anchor2$1@-1.47788 ];

[ anchor2$2@-0.95048 ];

[ anchor2$3@-0.04921 ];

[ anchor2$4@0.90495 ];

[ anchor3$1@-1.42442 ];

[ anchor3$2@-0.70078 ];

[ anchor3$3@-0.14289 ];

[ anchor3$4@0.39413 ];

[ gamesmap$1@-0.84597 ];

[ gamesmap$2@-0.34027 ];

[ gamesmap$3@0.24233 ];

[ gamesmap$4@0.87548 ];

[ museummap$1@-1.19910 ];

[ museummap$2@-0.51771 ];

[ museummap$3@0.44360 ];

[ museummap$4@1.03124 ];

[ readamap$1@-0.80050 ];

[ readamap$2@0.10043 ];

[ readamap$3@1.19862 ];

[ readamap$4@2.07734 ];

[ libmap$1@-0.52670 ];

[ libmap$2@0.33757 ];

[ libmap$3@1.14129 ];

[ libmap$4@1.96673 ];

[ lettermap$1@-2.40458 ];

[ lettermap$2@-0.98413 ];

[ lettermap$3@0.38072 ];

[ lettermap$4@1.40285 ];

[ concertmap$1@-1.63403 ];

[ concertmap$2@-1.11455 ];

[ concertmap$3@-0.28740 ];

[ concertmap$4@0.25617 ];

[ gamesmars$1@-0.84597 ];

[ gamesmars$2@-0.34027 ];

[ gamesmars$3@0.24233 ];

[ gamesmars$4@0.87548 ];

[ museummars$1@-1.13633 ];

[ museummars$2@-0.28781 ];

[ museummars$3@0.69920 ];

[ museummars$4@1.23144 ];

[ readamarsaa$1@-0.24986 ];

[ readamarsaa$2@0.72113 ];

[ readamarsaa$3@1.65171 ];

[ readamarsaa$4@2.46766 ];

[ libmarsaa$1@0.18023 ];

[ libmarsaa$2@0.96348 ];

[ libmarsaa$3@1.67621 ];

[ libmarsaa$4@2.31657 ];

[ lettermarsaa$1@-2.21042 ];

[ lettermarsaa$2@-0.54588 ];

[ lettermarsaa$3@0.70659 ];

[ lettermarsaa$4@1.50129 ];

[ concertmarsaa$1@-1.55068 ];

[ concertmarsaa$2@-0.94556 ];

[ concertmarsaa$3@0.00998 ];

[ concertmarsaa$4@0.57376 ];

[ gamesros$1@-0.84597 ];

[ gamesros$2@-0.34027 ];

[ gamesros$3@0.24233 ];

[ gamesros$4@0.87548 ];

[ museumros$1@0.59206 ];

[ museumros$2@2.09186 ];

[ museumros$3@2.68148 ];

[ museumros$4@3.32148 ];

[ tvros$1@-1.71601 ];

[ tvros$2@-1.51051 ];

[ tvros$3@-1.18412 ];

[ tvros$4@-0.61010 ];

[ radioros$1@-0.88267 ];

[ radioros$2@-0.70875 ];

[ radioros$3@-0.48086 ];

[ radioros$4@-0.13132 ];

CogAct1*1;

[CogAct1*0];
